# Application of association rules to ball possessions in professional men’s football

**DOI:** 10.3389/fpsyg.2025.1527437

**Published:** 2025-06-13

**Authors:** Rubén Maneiro, Mario Amatria, José L. Losada, Gudberg K. Jonsson, Antonio Ardá, Iyán Iván-Baragaño

**Affiliations:** ^1^Faculty of Science Education and Sport, University of Vigo, Vigo, Spain; ^2^Faculty of Science Education, Pontifical University of Salamanca, Salamanca, Spain; ^3^Department of Social Psychology and Quantitative Phycology, University of Barcelona, Barcelona, Spain; ^4^Human Behavior Laboratory, School of Health Sciences, University of Iceland, Reykjavík, Iceland; ^5^Department of Physical and Sport Education, University of A Coruña, A Coruña, Spain; ^6^Department of Sports Sciences, Faculty of Medicine, Health and Sports, European University of Madrid, Madrid, Spain

**Keywords:** performance analysis, football, soccer, association rules, observational methodology

## Abstract

**Introduction:**

This study represents one of the first attempts to apply association rule mining to the analysis of ball possession in professional men’s football. The goal was to uncover hidden patterns among key tactical variables influencing possession success.

**Methods:**

Using observational methodology, 2,324 ball possessions from the UEFA Euro 2020 championship were analyzed. The Apriori algorithm was applied to generate a total of 4,818 association rules, focusing on variables such as possession time, tactical intent, and field zones where possession occurred.

**Results:**

The results show that short possessions, with the intent to progress and developed in advanced zones of the field, are strongly associated with successful outcomes. This is reflected in high lift values (up to 40) and strong confidence levels. In contrast, long possessions in offensive zones did not consistently correlate with success.

**Discussion:**

These findings suggest that possession duration alone is not a reliable predictor of success. Instead, the combination of short possessions, in advanced zones, and with progressive intent, is more closely associated with positive outcomes. Association rule mining emerges as a valid and interpretable tool to support decision-making in elite football.

## 1 Introduction

A key aspect that differentiates performance analysis from other branches of Sports Sciences is its focus on studying athletes’ actual behavior in their habitual environments, such as during competitions or training sessions (O’Donoghue, 2014). Traditionally, this evaluation has been conducted through observation, either in real time or by reviewing recordings with automatic devices and electronic models. Thus, performance analysis seeks to objectively, systematically, and specifically record the spontaneous behaviors of players and teams in context. By properly coding and analyzing these behaviors, it generates valid and useful results for advancing knowledge in the field.

In high-performance football, performance analysis is a relatively young scientific discipline (González et al., 2018). Despite significant progress in recent years, it has yet to reach full scientific maturity or provide coaches with precise information to enhance decision-making. Two macro-stages can be clearly distinguished in this field: an initial descriptive phase, where studies aimed primarily at documenting and explaining events during competition (McGarry, 2009); and a subsequent comparative and predictive stage, which incorporates theoretical models to anticipate behaviors within the game context (Memmert and Rein, 2018).

At the end of the 20th century and the beginning of the 21st, the dominant method for describing performance was “quantitative assessment,” based on frequency calculations and event descriptions. For instance, studies such as Barros et al. (2007) analyzed distances covered by different players, while Dellal et al. (2011) examined technical performance. Wallace and Norton (2014) characterized gameplay based on execution speed and various technical patterns, and Gréhaigne et al. (2001) explored playing space and player movement zones from a qualitative perspective.

With the integration of new statistical and technological tools, the vast amount of data generated in competitions necessitated more sophisticated analysis approaches. This led to the use of association and independence analyses to determine relationships between key variables. For example, Oliva-Lozano et al. (2023) identified that the first 15 min of each half produced the highest frequency of maximum-speed actions; Martínez-Hernández et al. (2023) found associations between goal-scoring and prior movements; and Clemente et al. (2023) established links between coaches’ nationalities and training methods. A particularly notable study by Teoldo and Cardoso (2021) demonstrated a statistical relationship between a player’s birth month and the likelihood of becoming a professional footballer. This statistical development has led to a significant advance in football performance research, uncovering associations between categorical variables that were previously difficult to quantify.

The emergence of “dynamic tactical assessments” or “match analysis 4.0” (Memmert and Rein, 2018) in 2011 marked a new phase, incorporating advanced techniques such as machine learning. These approaches enable predictive modeling of in-game events Cohen (1960). Nunes et al. (2020) using GPS data found that larger playing areas with a high number of players involved promoted high-intensity movement, while smaller areas allowed to reduce the pace of play, in addition to facilitating more technical actions such as dribbling, blocking or interceptions; in another study of a similar nature (Nunes et al., 2021) they showed that playing in situations of high inferiority (such as 4vs2) can increase the physical demand of the team in numerical inferiority; Iván-Baragaño et al. (2024) analyzed the evolution of technical and tactical aspects in women’s football, and Immler et al. (2021) examined coaching styles. Wright et al. (2011) applied binary logistic regression to demonstrate that goalkeeper positioning and shot type are directly associated with goal outcomes.

Given these advancements, it is clear that analytical techniques have driven a paradigm shift in football science. However, despite their widespread adoption among researchers, continued innovation remains crucial. One underexplored technique is association rule mining, a key method in data mining (Han and Fu, 1995; Han and Pei, 2000) used to identify hidden relationships within large datasets (Imielinski, 1996). Association rules, also known as affinity rules, uncover frequent patterns that co-occur, offering valuable insights for decision-making.

Association rule mining is based on three fundamental measures: support, confidence, and lift. Support refers to the proportion of instances where two or more elements appear together (e.g., a transaction contains X, Y, and Z). Confidence measures the conditional probability that an item appears given the presence of another (e.g., if X and Y occur, Z is also likely to occur). Lift evaluates the strength of this relationship relative to random chance: Lift > 1 indicates a positive association (items co-occur more often than expected by chance), Lift < 1 indicates a negative association, and Lift = 1 suggests no association. These metrics enable the identification of meaningful patterns that can inform tactical and strategic decision-making.

In sports research, association rule mining has been scarcely applied (Stein et al., 2017), despite its potential for analyzing chaotic and unpredictable environments like football. Given the dynamic interactions between players, examining large datasets can reveal crucial tactical combinations that influence match outcomes. By identifying recurrent patterns in play sequences, coaches can optimize their strategies against various opponents. This is particularly valuable in football, where minor tactical adjustments can yield significant advantages. Furthermore, association rules can aid in anticipating opposing teams’ strategies, providing a data-driven competitive edge.

Considering the above, applying association rule mining to team sports analysis is a promising and methodologically sound area of research. Investigating temporal associations in football data could enhance our understanding of tactical dynamics, leading to scientifically backed recommendations for performance optimization. Consequently, this study aims to analyze possession-based interactions in high-performance football, identifying recurring patterns and systematic behaviors to contribute to the growing body of knowledge in this field.

## 2 Materials and methods

### 2.1 Design and participants

For the development of this study, the observational methodology was used (Anguera, 1979), a methodology that has proven to be one of the most suitable for studying the spontaneous interaction behavior among athletes, including its mixed-methods approach (Castañer et al., 2013). The design of this research is punctual—intrasessional follow-up—multidimensional, and nomothetic (Anguera et al., 2011). It should be noted that the observation is governed by scientific criteria, with full observer perceptivity and a non-participant observer.

To select the participants, an intentional or convenience a convenience sampling method was used (Anguera et al., 2011). Ball possessions during the final phase of the UEFA EURO, specifically the 2020 edition, were collected and analyzed. In total, 2,324 ball possessions were examined. The inclusion criteria proposed by Garganta (1997) were followed. Additionally, extra time periods were excluded, as they were considered special situations. The exclusion of extra time was based on ensuring homogeneity in the competitive context. In tournaments such as UEFA Euro 2020, not all matches include extra time, which introduces structural variability that affects data comparability. Extra time is generally influenced by situational factors such as accumulated fatigue, strategic game management, or the likelihood of a penalty shootout. Therefore, its inclusion would have introduced contextual bias. However, it is also acknowledged that excluding extra time may omit critical moments that can affect match outcomes, potentially reducing the applicability of the findings.

Data collection was conducted using publicly available footage broadcast on television, of general interest and sponsored by various private entities.

### 2.2 Observational instrument

To carry out this work, the observational instrument proposed by Maneiro et al. (2020) (Table 1) was used, given its effective molar-molecular fit in collecting this type of data, as demonstrated in similar studies on men’s football. The observational instrument is a combination of field format and category systems (Anguera et al., 2007), being nested within the various field formats.

**Table 1:** Observation instrument.

	Criteria	Categories	Description
1	Half time (match part)	First half Second half	This criterion refers to whether the possession occurs in the first or second half of the match.
2	Start form	Transition Set piece	This criterion refers to whether the possession begins in transition (with the ball in play) or through a set-piece action.
3	(COI) Interaction context	AR: forward versus delayed line AM: forward versus middle line AA: forward versus forward line MM: middle versus middle line MR: middle versus delayed line MA: middle versus forward line RA: delayed versus forward line RM: delayed versus middle line PA: goalkeeper versus forward line	This criterion refers to which line recovers the ball and against which opposing line: AR (forward vs. delayed line), AM (forward vs. middle line), AA (forward vs. forward line), MM (middle vs. middle line), MR (middle vs. delayed line), MA (middle vs. forward line), RA (delayed vs. forward line), RM (delayed vs. middle line), PA (goalkeeper vs. forward line).
4	Intention	Progress	This criterion refers to the tactical intention of the team: to progress (when the team clearly and intentionally advances toward the opponent’s field by making at least two forward passes or sending the ball to the penalty area), or to stay (when the team clearly and intentionally does not advance with the ball, plays laterally, or the first two passes are made backward).
		Keep	
5	MD		This criterion refers to the time the observed team maintains ball possession in its defensive zone.
6	MO		This criterion refers to the time the observed team maintains ball possession in its offensive zone.
7	ZC		This criterion refers to the zone in which the team maintained possession for the longest time.
8	Time possession		This criterion refers to the total duration of ball possession.
9	Passes		This criterion refers to the total number of passes made by the team in possession of the ball.
10	Move outcome	Goal scored	This criterion refers to the outcome of the ball possession: goal (goal scored), shot (shot on goal), sending to the area (ball sent into the opponent’s area), or no success (no significant outcome).
		Shot on goal	
		Send to area	
		No success	
11	Match status	Winning Drawing Losing	This criterion refers to the partial match status at the time of possession (winning, drawing, or losing).
12	Final score	Win Draw Lose	This criterion refers to the final result of the match for the team in possession (win, draw, or loss).

Source: Maneiro et al. (2020).

### 2.3 Registration and coding

The data recording (Hernández-Mendo et al., 2014) was conducted using the Lince Plus program (Soto et al., 2019). Inter-observer agreement was analyzed by pairs, generating all six possible combinations between the four observers (Ob1–Ob2, Ob1–Ob3, Ob1–Ob4, Ob2–Ob3, Ob2–Ob4, and Ob3–Ob4). The average Kappa value obtained was 0.92, which is classified as very good according to the scale proposed by Fleiss et al. (2003). Four observers were selected for data collection, all of whom hold doctorates in Sports Sciences and are UEFA PRO-licensed coaches. Additionally, to ensure the quality of the methodological process, a methodologist expert in observational methodology also participated in the study. Although formal blinding was not implemented, observer bias was minimized through strict adherence to standardized training protocols, including eight preparatory sessions, individual calibration phases, and collective discussion of discrepancies.

Before the coding process and to reduce variability among observers, eight training sessions were conducted, following Anguera et al. (1999). Each training session lasted 2 h. The first three sessions were conducted in a group with the selected observers. During these sessions, the study was presented theoretically, player behaviors to be observed were defined, the observational instrument was introduced, and the observers were trained in using the Lince Plus recording tool. In the fourth session, observers participated in observing and recording 20 pre-selected offensive actions, organized from least to most complex by the principal investigator. After recording the actions, discrepancies were discussed. The fifth and sixth sessions were conducted individually with each observer. The initial delimitation of the recorded actions was performed by the principal investigator, and observers received instruction on how to record the actions. The last two training sessions were also conducted individually, during which Cohen’s Kappa coefficient of agreement was verified between the principal investigator and each observer. Ten percent of the total sample (*n* = 233) was used to assess data quality. Although formal blinding was not implemented, observer bias was minimized through strict adherence to standardized training protocols, including eight preparatory sessions, individual calibration phases, and collective discussion of discrepancies. This methodological rigor ensured consistency and objectivity in the coding process.

### 2.4 Data analysis

For statistical analysis, R version 4.3.1 was used with the libraries arules, arulesViz, and RColorBrewer. Specifically, the arules package (version 1.7–7), titled *Mining Association Rules and Frequent Itemsets*, was used. The URL is https://cran.r-project.org/src/contrib/Archive/arules. The arulesViz package (version 1.5–2), titled *Visualizing Association Rules and Frequent Itemsets*, was also used (URL: https://cran.r-project.org/web/packages/arulesViz). Finally, the RColorBrewer package (version 1.1–3) was used (URL: https://cran.r-project.org/web/packages/RColorBrewer/). Association rules, a branch of artificial intelligence (AI), were employed to identify general “if-then” patterns, applying specific criteria to define key relationships. This type of analysis does not test causation but simply identifies temporal associations. Nevertheless, it is useful for establishing hypotheses that can later be analyzed in greater depth. Additionally, it does not rely on correlation and does not imply causation. Within a given timeframe, once an event is associated with another, this relationship may vary on different occasions.

It is an unsupervised learning technique used to extract relevant information from large datasets. Each association rule is linked to various numerical measures that determine its relevance. Its primary strength lies in interpretability, which is increasingly valued in the field of machine learning. The basic concepts of association rules include items, understood as the elements that make up a transaction, and itemset, defined as a set of elements within a transaction.

Measures such as *support*, *confidence*, and *lift* are used. *Support* indicates the popularity of an item, measured by the proportion of transactions in which a set of items appears. *Confidence* indicates the probability that item Y occurs when item X has already occurred, expressed as {X = > Y}. This is measured by the proportion of transactions with item X in which item Y also appears. Finally, *lift* is the ratio between the observed support and what would be expected if X and Y were independent (Table 2).

**Table 2:** Formula of the association rules indicators.

Support (X= > Y) = $\frac{\text{Number of transactions containing both}\;X\;\text{and}\;Y}{\text{Total number of transactions}}$
Confidence (X= > Y)= $\;\frac{\text{Number of transactions containing both}\;X\;\text{and}\;Y.}{\text{Number of transactions containing}\;X}$
Lift (X= > Y)= $\frac{\text{Confidence}\;(X=>Y)\;}{\text{Support}\;(Y)}$

In the context of football, lift values above 1.2 may indicate meaningful associations when accompanied by sufficient support and confidence. Values exceeding 1.5 can be considered tactically relevant, while lift values over 2 point to strong associations far from randomness (Stein et al., 2017).

The structure of the association rules (i.e., the allocation of antecedents and consequents) was determined automatically by the Apriori algorithm based on item frequency and co-occurrence, subject to minimum support and confidence thresholds. However, the selection and codification of the input variables were theory-driven and based on domain relevance in football performance analysis. In practice, this means that while Apriori algorithmically generated the rules, the set of possible items was predefined through a observational instrument.

This manuscript retains the notation = > to represent the relationship between antecedents and consequents in association rules, in line with the output format of the arules package in R, which is widely used in data mining.

Subsequently, the “*A priori*” algorithm is used, which leverages prior knowledge of frequent properties within the dataset. It is applied to identify frequent itemsets.

## 3 Results

**Figure 1:**
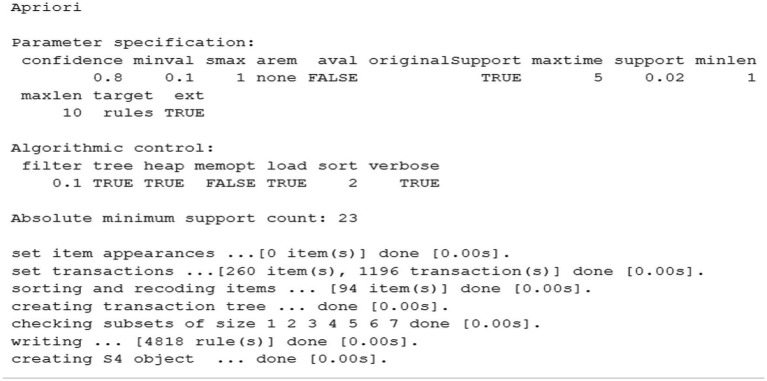
Application of the Apriori algorithm. Schematic representation of the application of the Apriori algorithm in this study. The diagram outlines the analytical workflow from the coding of ball possessions to the generation of 4,818 association rules, specifying the parameters used (minimum support of 2%, minimum confidence of 80%, and a maximum of 10 items per rule). It also illustrates the transformation of data into transactions and the rule selection process.

The “A priori” algorithm is applied (Figure 1), and the fundamental qualitative measures are obtained to identify the association rules. The items used are: HalfTime, StatForm, COI, Intention, MD, MO, ZC, Time Possession, Passes, Move Outcome, MatchStatus, and Final Score.

The output corresponds to the execution of the Apriori algorithm in R for association rule mining, using the arules package. The main parameters are specified: minimum confidence level of 80%, minimum support of 2%, and a maximum of 10 generated rules. The dataset includes 1,196 transactions and 260 items, of which 94 were recoded to optimize the analysis. The transaction tree structure is successfully created, verifying subsets of size 1 to 7. As a result, 4,818 association rules were generated, with a minimum absolute support threshold of 23 transactions. Algorithmic controls such as filtering, sorting, and memory storage are applied. Finally, the process concludes with the creation of the S4 object that stores the obtained rules.

**Figure 2:**
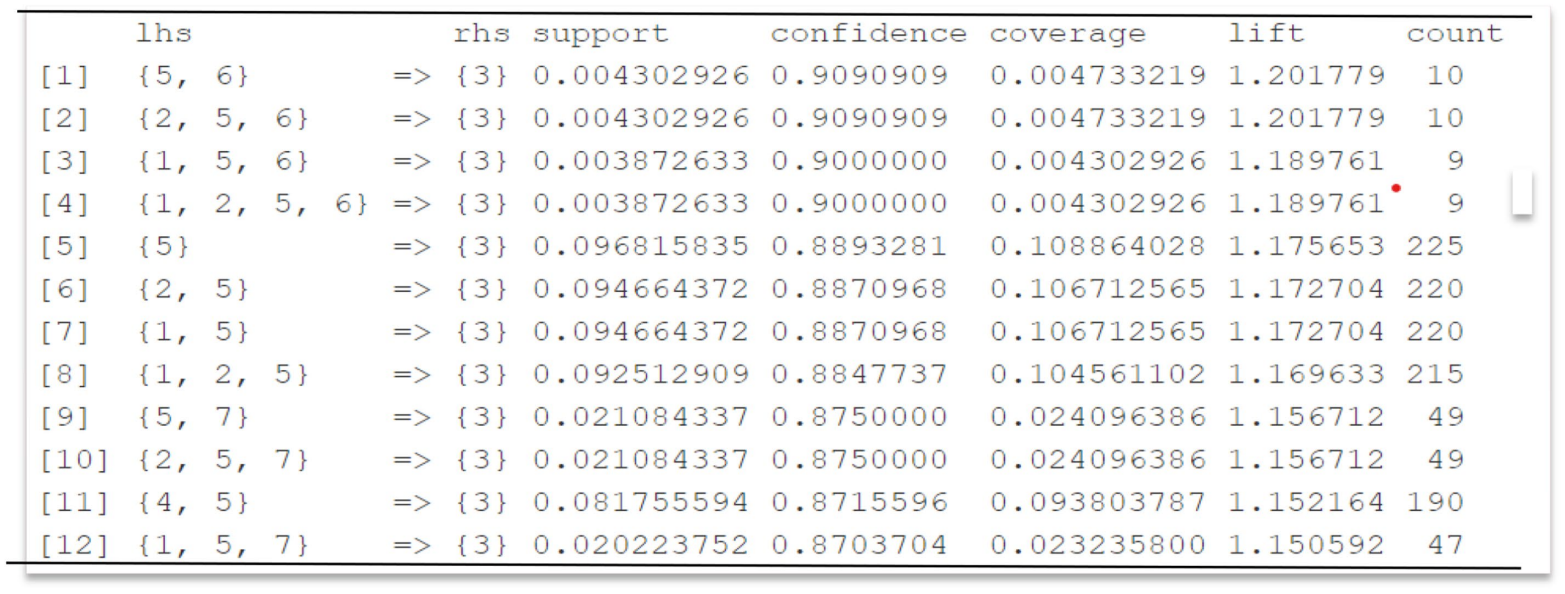
Qualitative measures. Visualization of the qualitative measures derived from the Apriori algorithm. The specific support, confidence, and lift values that characterize the selected rules are shown. These metrics allow for the identification of relevant associations between tactical variables, and are key criteria for selecting the most significant rules in the analysis. The coding in the LHS column corresponds to the variables collected in the observation tool (Table 1), following the same numerical order of presentation.

In this case, a minimum support of 0.02, a minimum confidence of 0.8, and a number of elements between 1 and 10 were set to generate association rules. Under these conditions, 4,818 rules were obtained and then subjected to a pruning process to improve clarity and analytical value. Specifically, four steps were followed: (1) elimination of redundant or semantically overlapping rules, given the high dimensionality of the dataset (260 items derived from 1,196 transactions); (2) application of a secondary filter prioritizing rules with a lift > 1 to ensure stronger-than-random associations; (3) contextual filtering to remove rules lacking football-specific tactical relevance; (4) final selection based on lift and support values to retain the most interpretable and meaningful patterns. Figure 2 presents the final result, where the specific values of support and confidence may exceed the initially defined minimum thresholds.

The criterion used to select association rules in this case is a lift greater than 1, indicating that the items are positively related.

Therefore, the first twelve rules meet this requirement. In this study, we selected the first two rules showing the highest values. The first association rule links the items MD and MO with the item COI, with a lift of 1.20. The second association rule links the items StartForm, MD, and MO with the item COI, also with a lift of 1.20.

The established relationship includes the Start Form variable, which can occur either through a ball steal (a change in ball possession from one team to another via a turnover) or a regulatory incident (such as a set play). Once the ball is recovered (Start Form), possession begins in the team’s own half (MD), after which they can progress and establish possession in the opponent’s half (MO). As with the previous scenario, the longer the possession lasts, the higher the chances for the various lines or interaction contexts to emerge.

**Figure 3:**
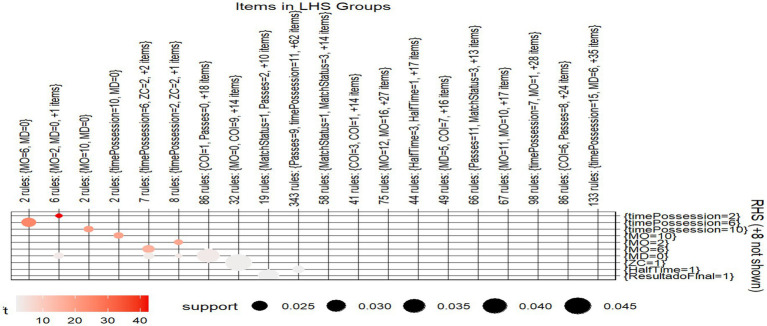
Grouped matrix of 4818 rules. Grouped matrix of 4,818 association rules generated using the Apriori algorithm. The horizontal axis represents the sets of antecedents (lhs), and the vertical axis shows the consequents (rhs). Each circle represents a rule, with its size indicating support (relative frequency) and its color indicating the lift value (strength of association). More intense colors reflect stronger associations between items.

A 2D graphical visualization (Figure 3) displays 4,818 association rules using color-coded bubbles: darker colors represent higher Lift values (stronger relationships), and larger bubble sizes indicate higher Support (frequency). This visual format helps to easily interpret the relationships between antecedents and consequents.

Three main groups of rules stand out:

- 15 rules include MD = 2 and ZC = 2, plus two other items, predicting that “time of possession” will be 2. These rules have high Lift values, indicating strong associations, and are shown with darker bubbles.
- 8 rules include MO = 6 and MD = 0, plus two additional items, predicting a “time of possession” of 6. Although fewer, these rules also show strong associations due to their high Lift.
- 3 rules include MO = 10 and MD = 0, with one more item, predicting a “time of possession” of 10. Despite being the smallest group, they are very relevant because of their strong Lift values.

These three groups were identified computationally during rule generation and are represented in Figure 3 as visually clustered bubbles with similar support and lift characteristics. Although individual rules are not labeled in the figure, their grouping is reflected through bubble proximity and color.

Notably, some rules in the dataset reach extremely high Lift values (above 30 or even 40) which, in the context of association rule mining, indicate exceptionally strong and non-random associations. In football terms, such high lift suggests that the co-occurrence of certain tactical elements (in this case, the teams’ tactical objective during ball possession is to advance toward the opponent’s goal, that is, they seek to implement building strategies that allow vertical progression toward the opposing goal) is far more likely than expected by chance, revealing stable and recurrent patterns in high-performance play.

This visualization, based on Support and Lift, clearly highlights the most relevant patterns in the dataset, making the analysis more intuitive and easier to interpret. The tactical aim of teams during ball possession is to advance toward the opponent’s goal, meaning they seek to implement building strategies that enable vertical progression toward the opposing goal.

On the other hand, the rule with the highest Support comprises 412 rules formed by the antecedents COI = 1 and Passes = 0, with MD = 0 as the consequent, along with 22 additional items. Although this rule is not the focus of analysis, it provides insight into the items with the highest frequency.

## 4 Discussion

This study was conducted with the objective of analyzing possible relationships established during ball possessions in high-performance football, aiming to identify regularities or general behavior patterns. To achieve this goal, a novel statistical technique in the field of sports performance (association rules) was implemented.

Firstly, the algorithm generated 4,818 association rules across 2,324 ball possessions (Figure 1), indicating a high volume of patterns or temporal associations. These associations suggest the existence of hidden relationships among the variables considered during ball possessions that frequently appear in matches. Football is a sport characterized by collaboration and opposition, shared space and simultaneous participation, where different game structures at the micro (1vs1, 2vs2), meso (3vs3, 4vs4, 5vs5.) and macro (11vs11) levels are interconnected and can behave as superorganisms (Duarte et al., 2012). Performance in these team sports arises from interactions, where the actions of a player or group of players are a result of interpersonal relationships between teammates and opponents (Santos et al., 2018). Furthermore, these interactions between players can give rise to both individual and collective technical behaviors, such as sprints, dribbles, blocks, or even promoting variability in the number of passes.

On the other hand, in Figure 2, the available data show that the relationship between the variables MD, MO = > COI indicates that these elements appear together more frequently than they would by chance, establishing a positive relationship. The criterion used for selecting the association rules in this case is a lift greater than 1. This signifies a positive relationship between the antecedent and the consequent, with a lift of 1.20 alongside high confidence and support. The RHS (Right-Hand Side) shows that in this combination of associations in football, the variable COI is always present. Specifically, in football terms, it can be asserted that increased possession time in either offensive or defensive sectors correlates with more associations occurring within particular lines or interaction contexts (Castellano, 2000). This confirms that teams establish connections among the different lines set up tactically (Kannekens et al., 2009; Wiemeyer, 2003). Regarding the possession zone, while not explicitly concluded from these results, we may predict that longer possession time correlates with a higher number of goals and overall success (Casal et al., 2017; Collet, 2013).

To aid the tactical interpretation of the MD, MO = > COI rules, it is important to clarify that MD refers to the time of possession in the defensive midfield, MO to the time of possession in the offensive midfield, and COI to the context of interaction between the recovering line and the opposing line. Thus, a rule with MD and MO as antecedents and COI as the consequent—especially with a lift greater than 1—suggests that maintaining possession across both midfield zones is frequently linked to specific interaction dynamics. This implies coordinated progression behavior across lines, which has tactical relevance. Moreover, high lift values (some exceeding 30 or 40) reflect extremely strong and non-random associations between tactical events. In football terms, a lift of 2 already indicates that the co-occurrence of two elements is twice as likely as by chance, and values above 1.5 may be considered tactically meaningful when supported by adequate confidence and support levels.

From the same figure, a connection between the variables Start Form, MD, and MO = > COI is also evident, showing a lift of 1.20. This relationship involves the variable Start Form, which can result from a ball recovery (a turnover where possession shifts from one team to another) or a regulatory event (such as a set piece). Following a ball recovery (Start Form), possession typically begins in the team’s own half (MD) before transitioning into the opponent’s half (MO). Similar to the previous case, an extended possession duration is associated with a higher likelihood of interactions across different tactical contexts. This occurs as the team progresses toward the opponent’s goal in pursuit of scoring, necessitating coordinated actions among teammates and defensive responses from the opposing side (Maneiro et al., 2019; González-Ródenas et al., 2021).

The grouped matrix presented in Figure 3 also reveals interesting results that align with the previous ones. The association with the highest intensity consists of 6 rules with MO = 2 and MD = 0, and is always accompanied by RHS equal to 2, corresponding to the “time possession” variable, with a high lift of 40. The second most interesting association appears in the first column, with 2 rules having MO = 6 and MD = 0. Again, these results confirm the earlier findings about the strong association between possession time in both defensive and offensive midfield and total possession time, but this time with a strong association evident from the lift value (30). This allows us to state that possession time in either of the two areas of the field determines total possession time, data that aligns with previous work on possession analysis in high-performance football (Link and Hoernig, 2017).

Although the present study applied association rules to discover frequent and significant patterns in ball possession sequences, it is important to situate these findings within the broader landscape of methods used in soccer performance analysis. Previous research has employed techniques such as regression analysis to identify and categorize playing styles in elite teams (Fernandez-Navarro et al., 2018), as well as to predict outcomes based on performance indicators (Liu et al., 2013). On the other hand, unsupervised clustering techniques and big data approaches have been used to identify situations and behaviors in football matches, allowing for a more detailed segmentation of game dynamics (Michael et al., 2018; Rein and Memmert, 2016). Compared to these methods, association rules offer an alternative perspective, by highlighting non-linear and multivariate co-occurrence patterns that may go unnoticed in models that require predefined outcomes or assume independence between variables. Therefore, this study contributes to the field by introducing an approach that allows detecting emergent tactical structures from combinations of frequent items, thus enriching the set of methodological tools available in the analysis of football performance.

Beyond post-match analysis, the association patterns identified in this study could be applied in real-time tactical decision-making. For instance, if during a match the coaching staff observes repeated sequences involving short possessions initiated in the offensive midfield and ending in the attacking third, they could recognize this as a favorable pattern previously linked to effective outcomes. This awareness could inform on-the-fly decisions such as adjusting pressing intensity, modifying player roles, or reinforcing vertical attacking transitions. These insights could also support pre-match planning, helping coaches design training drills that replicate high-impact patterns identified through association rules, thereby bridging data analysis with applied tactical practice.

At the applied level, and from a coaching recommendation perspective, the patterns identified through association rules can be valuable tools for coaches in different training contexts. In task design, the most frequent and strongly associated behaviors, such as short possessions initiated in attacking midfield with the intent of progressing, can be used to structure drills that replicate these successful sequences. Regarding opponent analysis, coaches and analysts can look for recurring possession patterns employed by the opponent and compare them with the favorable or unfavorable rules identified in this study, thereby adjusting the match plan.

Despite its contributions, the present study has limitations. Association rule mining does not infer causality, and interpreting a large number of generated rules can be complex. Additionally, the analysis was based exclusively on a single tournament (UEFA Euro 2020), which limits the generalizability of the findings. Applying this method to other competitions—such as national leagues or World Cups—would help validate and expand the applicability of the approach. Moreover, the inclusion of extra time was excluded to maintain contextual consistency, although this may omit decisive phases of the match. The exclusion was based on structural heterogeneity: not all matches involve extra time, and when it does occur, it is heavily influenced by fatigue, strategic management, or the likelihood of penalty kicks. Nevertheless, this may reduce the ecological validity of the analysis.

Furthermore, several contextual variables were not included in the model, such as individual player actions, referee decisions, crowd influence, or environmental conditions. Future research should integrate these factors to enrich the complexity and ecological validity of tactical analysis. Also, it would be relevant to explore how individual possessions (e.g., by player roles or positions) interact within collective sequences. In addition, association rule mining could be applied to different formations or opponent strategies, and combined with other machine learning methods for predictive modeling. Finally, we encourage future research to explore the application of this method in women’s football, contributing to the growing field of gender-informed performance analysis.

## 5 Conclusion

This study highlights the usefulness of association rule mining in analyzing ball possession patterns in high-performance football. The application of this method allowed for the identification of frequent, interpretable, and tactically relevant relationships between contextual variables, offering a detailed view of how certain combinations of play behaviors tend to co-occur.

The technique proved to be valuable due to several strengths: a high volume of generated rules (4,818), internal consistency across clusters of rules, and alignment with well-established tactical principles—such as short possessions initiated in the offensive midfield with the intention to progress. These factors support the validity of association rules as a method for capturing meaningful patterns, and their reliability is reinforced by the recurrence of consistent rule groups throughout different combinations of antecedents and consequents.

## Data Availability

The raw data supporting the conclusions of this article will be made available by the authors, without undue reservation.
